# Pilot study to investigate the effect of long-term exposure to high *p*CO_2_ on adult cod (*Gadus morhua*) otolith morphology and calcium carbonate deposition

**DOI:** 10.1007/s10695-021-01016-6

**Published:** 2021-09-28

**Authors:** Clara Coll-Lladó, Felix Mittermayer, Paul Brian Webb, Nicola Allison, Catriona Clemmesen, Martina Stiasny, Christopher Robert Bridges, Gwendolin Göttler, Daniel Garcia de la serrana

**Affiliations:** 1 Scottish Oceans Institute, School of Biology, University of St Andrews, Scotland, UK; 2 Marine Evolutionary Ecology, GEOMAR Helmholtz Centre for Ocean Research Kiel, Kiel, Germany; 3 School of Chemistry, University of St Andrews, Scotland, UK; 4 School of Earth and Environmental Sciences, University of St Andrews, Scotland, UK; 5 Ocean and Earth Science, National Oceanography Center Southampton, University of Southampton, Waterfront Campus, Southampton, UK; 6 Institut Für Stoffwechselphysiologie/AG Ecophysiology, Heinrich-Heine-Universität Düsseldorf, Düsseldorf, Germany; 7 Department of Cell Biology, Physiology and Immunology, Faculty of Biology, University of Barcelona, Barcelona, Spain

**Keywords:** Adult cod, Calcite, Gender, Otolith, Ocean acidification

## Abstract

To date the study of ocean acidification on fish otolith formation has been mainly focused on larval and juvenile stages. In the present pilot study, wild-captured adult Atlantic cod (*Gadus morhua*) were exposed to two different levels of *p*CO_2,_ 422µatm (ambient, low *p*CO_2_) or 1091µatm (high *p*CO_2_), for a period of 30 weeks (from mid-October to early April 2014–2015) in order to study the effects on otolith size, shape and CaCO_3_ crystallization amongst other biological parameters. We found that otoliths from cod exposed to high *p*CO_2_ were slightly smaller (− 3.4% in length; − 3.3% in perimeter), rounder (− 2.9% circularity and + 4% roundness) but heavier (+ 5%) than the low *p*CO_2_ group. Interestingly, there were different effects in males and females; for instance, male cods exposed to high *p*CO_2_ exhibited significant changes in circularity (− 3%) and roundness (+ 4%) compared to the low *p*CO_2_ males, but without significant changes on otolith dimensions, while females exposed to high *p*CO_2_ had smaller otoliths as shown for length (− 5.6%), width (− 2%), perimeter (− 3.5%) and area (− 4.8%). Furthermore, while the majority of the otoliths analysed showed normal aragonite deposition, 10% of fish exposed to 1091µatm of *p*CO_2_ had an abnormal accretion of calcite, suggesting a shift on calcium carbonate polymorph crystallization in some individuals under high *p*CO_2_ conditions. Our preliminary results indicate that high levels of *p*CO_2_ in adult Atlantic cod might affect otolith growth in a gender-specific way. Our findings reveal that otoliths from adult cod are affected by ocean acidification, and we believe that the present study will prompt further research into this currently under-explored area.

**Supplementary Information:**

The online version contains supplementary material available at 10.1007/s10695-021-01016-6.

## Background

Rising levels of atmospheric CO_2_ are leading to an increase in the average ocean CO_2_ partial pressure (*p*CO_2_), which translates to a decrease in environmental pH, a phenomenon known as ocean acidification (Caldeira 2005). Elevated *p*CO_2_ levels in sea water modify the saturation states of the different calcium carbonate (CaCO_3_) polymorphs (Jones et al. 2017) and alter the formation/dissolution rates of CaCO_3_-based structures (Hofmann et al. 2010). It is known that organisms with external calcium carbonate structures, such as mollusc shells, arthropod exoskeletons or coral “skeletons”, will be particularly sensitive to ocean acidification owing to the increase in carbonate dissolution rates that will weaken such structures (Hofmann et al. 2010). Otoliths are CaCO_3_-based structures located in the inner ear of fish and are responsible for hearing, balance and navigation (Popper et al. 2005). Otoliths are formed extracellularly through the accretion of CaCO_3_ in the form of aragonitic crystals, which are integrated into a protein-based matrix (Allemand et al. 2007). Otolith formation is a very dynamic process and varies significantly in response to environmental conditions (such as temperature or pH), fish ontology (such as age, gender, size or sexual maturation) and physiological status (nutrition, spawning or stress) (Morales-Nin 2000; Bestgen and Bundy 1998; Radtke and Fey 1996).

Research on the effects of ocean acidification on fish otolith formation has increased in recent years (Heuer and Grosell 2014) with some studies reporting an overall increase of otolith size and/or density for some species (Bignami et al. 2013; Checkley et al. 2009a; Maneja et al. 2013; Pimentel et al. 2014; Réveillac et al. 2015; Coll-Lladó et al. 2018). In addition, studies in gilthead sea bream (*Sparus aurata*) larvae have shown that exposure to 2000µatm of *p*CO_2_ for a period of 15 days also promotes the replacement of aragonite crystals (the common CaCO_3_ polymorph found in otoliths) by calcite in 21% of the individuals (Coll-Lladó et al. 2018). While calcite is a common CaCO_3_ polymorph in invertebrates, it is rarely found in fish otoliths, and only some primitive species of fish have calcite, combined with vaterite, as the main CaCO_3_ polymorph in their otoliths (Pracheil et al. 2017). Vaterite, but not calcite, otoliths are commonly found in many aquaculture-reared fish species (Gauldie et al. 1997; Whitley et al. 1999), and some authors have suggested that vaterite deposition is the result of abnormally high growth rates (Reimer et al. 2017) or high animal density in the farms (Austad et al. 2021). Functionally, transmission of sound waves through otoliths is significantly influenced by the size of the otolith and the presence of non-aragonite CaCO_3_ polymorphs, indicating that ocean acidification might have a negative impact on fish hearing and navigation if size and/or CaCO_3_ composition are affected (Bignami et al. 2013; Radford et al. 2021; Reimer et al. 2016). Despite advances in understanding the effects of ocean acidification on otolith formation, the vast majority of studies have been focusing on larval and juvenile stages.

Atlantic cod (*Gadus morhua*) is a commercially and ecologically important species with a wide distribution throughout the North Atlantic Ocean (Hylen et al. 2008) and considerable intraspecific stock-dependent differences in terms of growth rate, age at maturity, timing of spawning and life expectancy (Brander 2005). The majority of studies on otolith formation simulating ocean acidification conditions use relatively short exposure times to high *p*CO_2_ levels on fish embryos, larvae or juveniles (Checkley et al. 2009b; Coll-Lladó et al. 2018; Jarrold and Munday 2018; Réveillac et al. 2015), while experiments on adults are scarce. To understand how long-term exposure to *p*CO_2_ levels predicted for the year 2100 (Stocker 2013, appr. global average end of the century, RCP 8.5) would affect otolith growth and CaCO_3_ crystallization in adult North East Arctic Migratory Cod (Skrej), wild-captured adult fish were exposed to predicted (1091µatm; high *p*CO_2_) or ambient (422µatm; low *p*CO_2_) *p*CO_2_ levels for a period of 30 weeks, and otolith size, shape and main CaCO_3_ polymorph were thereafter analysed.

## Methods

### Ethics statement

This study was carried out in strict accordance with the laboratory regulations applicable in Norway. The application was approved by the National Regulatory Committee on the Ethics of Animal Experiments (Permit TRANSCOD project FOTS id 6915 and ACIDCOD project FOTS id 7346). All conditions and samplings were conducted to minimize suffering and stress.

As a result of the size of the fish used in the present work, and in order to ensure animal’s welfare, two adjacent tanks of 25m^3^ (25,000 L) were used with identical environmental conditions apart from the experimental treatment (low or high carbon dioxide) since replication of treatments were not possible.

### Fish husbandry and experimental conditions

Adult cods (*N* = 135) were caught in October 2014 in the Barents Sea at 70°15′ N, 19°00′ E and transported to NOFIMA’s Centre for Marine Aquaculture in Tromsø, Norway. The animals were sexed and measured before being equally distributed between the two 25m^3^ tanks with a continuous supply of water from the adjacent fjord. The CO_2_ was added to the header tank of the high CO_2_ experimental condition simulating a future climate scenario (Stocker 2013, global average end of the century, RCP 8.5), while no CO_2_ was added to the control tanks. From December 2014 to January 2015, a total of 20 animals from the low *p*CO_2_ and 13 from the high *p*CO_2_ died as a result of infections, 6 animals per group were sampled at the middle of the experimental period for other analyses, while the rest of animals (41 for low *p*CO_2_ and 49 for high *p*CO_2_) survived until being euthanized and sampled at the end of the experiment. In addition to total alkalinity (*A*_*T*_) and total carbon (*C*_*T*_), in situ *p*CO_2_ was estimated weekly (average values for the two treatments presented in Table 1, for more details of the methodology, see SI Stiasny et al. 2016). As a proxy for *p*CO_2_, the water pH (pH_NBS_) was continuously monitored using an IKS Aquastar Controller unit (Germany), which further controlled the CO_2_ inflow into the treatment tank. A handheld probe was used to measure the daily temperature, salinity and oxygen concentration. Water temperature and salinity were kept at ambient levels, while the light regime was adjusted to local sunrise and sunset on a weekly basis. In order to minimize any possible tank effect, experimental tanks were identical in structure and support systems and were placed 2 m of each other. All environmental conditions (light, temperature, salinity, etc.) except pH were the same during the whole experimental period. The fish were fed ad libitum with frozen capelin (*Mallotus villosus*) three times per week. Animals were caught with a landing net and euthanized using a MS222 overdose (200 mg/L) (tricaine methanesulfonate TMS, metacaine, finquel). All fish were sexed and weighed, and standard length (*SL*) measured for each individual. Otoliths were then extracted by cutting through the post parietal bones, removed with forceps and stored in labelled paper bags at room temperature until further analysis.

**Table 1:** Average sea water carbonate parameters during the experimental period

Treatment	T (C^o^)	C_T_ (µmol/L)	A_T_ (µeq L^−1^)	*p*CO_2_ (µatm)	pH_NBS_
Low-*p*CO_2_	4.6 ± 0.3	2154.9 ± 1.9	2285.9 ± 3.5	422.3 ± 4.3	8.02 ± 0.01
High-*p*CO_2_	4.6 ± 0.4	2274.7 ± 29.2	2288.2 ± 3.06	1091.5 ± 282.8	7.64 ± 0.10

*T* temperature, *C*_*T*_ total carbon, *At* total alkalinity, *pCO*_*2*_ CO_2_ partial pressure. Values are expressed as mean ± SD

### Otolith measurements

Right and left otoliths from individual animals (81 otoliths from the low *p*CO_2_ group and 98 otoliths for the high *p*CO_2_ group) were photographed using a digital camera fixed in a support (RS2 2 XA Copylizer, Kaiser Fototechnik, Germany) against a black background, homogeneous light and a millimetre scale for internal calibration. Otolith length (*OL*), width (*OR*), area (*OA*) and perimeter (*OP*) were measured twice using ImageJ software (Girish and Vijayalakshmi 2004) to ensure accuracy in the measurements (Supplementary File 1). Otolith total weight (*OW*) was measured using a digital scale (Sartorius, Germany, 0.01 g precision). For better comparison between otolith measurements from animals of different sizes, an adjusted means (least squares) was used for all measurements (see the statistics section below).

Otolith shape indexes, generally used for discrimination between stocks, were estimated based on previous literature (Pothin et al. 2006; Leguá et al. 2013) as follows:

$$Circularity \left(Cicl\right)=\frac{O{P}^{2}}{OA}$$

$$Roundness \left(Rd\right)=(4OA)/(\pi O{L}^{2})$$

$$Rectangularity \left(Rect\right)=OA/(OL*OR)$$

$$Ellipticity (Ellip)=(OL-OR)/(OL+OR)$$

### Raman spectroscopy

Right side otoliths were immersed in a 1% sodium hypochlorite solution for 10 min to remove all organic material on the surface followed by several washes with distilled water. Raman spectra were recorded from the otoliths surface with a Horiba Jobin Yvon LabRam HR instrument using 514 nm excitation wavelength and a 50 × magnification, long working distance objective. Laser intensity was attenuated using neutral density filters to prevent laser-induced transformation of the polymorph.

### Statistical analysis

All statistical analyses and graphs were conducted using R-Studio v.1.1.419 (RStudio Team 2015). Data normality and homogeneity of variance were estimated using the Shapiro–Wilk and Levene’s tests, respectively. A Box-Cox transformation approach was used to transform non-normally distributed data, and normality assumptions were estimated again. *OL*, *OR*, *OP* and *OA* measurements were analysed using linear mixed models (lme4 R-package) (Bates et al. 2015) with *treatment* as a fixed factor and *SL* and *gender* as covariants, while head side (*side*) was considered as random factor. When data was re-analysed for each gender, mixed models with *SL* as covariant and *side* as a random factor were used. Since animal body weight was highly correlated to *SL* (> 0.80 Pearson’s correlation) (Supplementary File 2), the parameter was not included in the model.

Shape indexes (*Cicl*, *Rect*, *Rd* and *Ellip*) are not directly affected by the length of the animal (SL was excluded from the statistical model), but they can be affected by the age of the animal. While empirical estimation of cod age by ring counting was not performed in the present pilot study, previous research has found that otolith weight (*OW*) correlates well with fish age (Bermejo 2014; Campana and Fowler 2012; Pawson 2006). Therefore, *OW* was used as a proxy for age and was included in the statistical model as covariant. Adjusted means (least squares) and standard errors (SE) for all parameters analysed were extracted from the mixed models using the *lsmeans* package (Russel 2016).

Unless otherwise indicated, values directly measured (raw data) are shown as mean ± SD, while adjusted values for the different parameters are shown as least squares ± SE. Pearson’s test was used to estimate the correlation between the different parameters. The signification threshold was established as *P*-value (*P*) < 0.05.

All graphs were produced using the ggplot2 R-build package (Wickham 2016). R-regression plots include 95% confidence intervals estimated using the *geom_smooth (method* = *"lm")* flag.

## Results

### Fish length and weight

The number of male cod in the present study was slightly higher than females in both low *p*CO_2_ (57% males) and high *p*CO_2_ (60% males) groups. The standard length (SL) measured and body weight at the end of the experimental period ranged from 68 to 105 cm and 4220 to 11400 g (Table 2). Animals from low *p*CO_2_ and high *p*CO_2_ groups had similar standard lengths (83.01 ± 5.71 and 86.19 ± 7.93 cm, respectively), while animals from the high *p*CO_2_ group were slightly heavier (7009 ± 1687 g) than from the low *p*CO_2_ group (6746 ± 1540 g) (*P* = 0.05). In both groups, female cods were significantly heavier (7656 ± 1790 g) and longer (87.25 ± 8.3 cm) than males (6693 ± 1422 g and 83.56 ± 5.94 cm), indicating sexual dimorphism (Table 3). Furthermore, although not significant, both males and females from the high *p*CO_2_ group were heavier (6745 ± 1492 and 7688 ± 1835 g) and longer (84.30 ± 6.6 and 89.24 ± 8.9 cm) compared to males and females from the low *p*CO_2_ group (6285 ± 1286 and 7180 ± 1720 g; 82.09 ± 4.67 and 84.23 ± 6.77 cm).

**Table 2:** Biometric data and otolith measurements in Atlantic cod

	Low *p*CO_2_ (*N* = 81)	High *p*CO_2_ (*N* = 98)	Diff (%)
*Bw (g)*	6746 ± 1540	7009 ± 1687	
*SL (cm)*	83.01 ± 5.71	86.19 ± 7.93	
*OW (mg)*	760 ± 130 (***0.78* ± *0.01***)*	800 ± 150 (***0.82* ± *0.01***)	+ 5
*OL (mm)*	20.06 ± 1.41 (***20.7* ± *0.10***)*	20.12 ± 1.45 (***20.0* ± *0.10***)	− 3.4
*OR (mm)*	9.85 ± 0.64 (***9.97* ± *0.06***)	10.07 ± 0.70 (***10.18* ± *0.06***)	+ 2
*OP (mm)*	51.76 ± 3.48 (***52.7* ± *0.33***)*	51.64 ± 3.32 (***51.0* ± *0.37***)	− 3.3
*OA (mm*^*2*^*)*	152.21 ± 19.30 (***154* ± *1.90***)	156.35 ± 19.21 (***157* ± *1.70***)	+ 1
*OD*	4.97 ± 0.40 (***5.01* ± *0.03***)	5.10 ± 0.45 (***5.13* ± *0.03***)	+ 2.5
*Cicl*	17.60 ± 1.40 (***17.7* ± *0.15***)*	17.06 ± 1.23 (***17.2* ± *0.17***)	− 2.9
*Rd*	0.48 ± 0.03 (***0.47* ± *0.003***)*	0.49 ± 0.03 (***0.49* ± *0.003***)	+ 4
*Rect*	0.77 ± 0.03 (***0.77* ± *0.003***)	0.77 ± 0.03 (***0.77* ± *0.003***)	+ 0
*Ellip*	0.34 ± 0.03 (***0.34* ± *0.003***)	0.33 ± 0.03 (***0.33* ± *0.004***)	− 3

Average values and adjusted means (in brackets) for Atlantic cod exposed to 429 (low *p*CO_2_) and 1091µatm (high_*p*CO_2_) for *Bw* body weight, *SL* standard length and *OW* otolith weight, *OL* otolith length, *OR* otolith radius, *OP* otolith perimeter, *OA* otolith area, *OD* = *OW/OA* otolith density, *Cicl* circularity, *Rd* roundness, *Rect* rectangularity and *Ellip* ellipticity. Unadjusted data is expressed as Mean ± SD and adjusted means as least squares ± SE. Differences (*Diff*) between adjusted means are expressed as a percentage. The number of individual otoliths analysed for each group is indicated (N)

Significant differences between groups for the *pCO*_*2*_ treatment are indicated with an asterisk when *P* < 0.05 (all *P*-values are in Supplementary File 3)

**Table 3:** Biometric data and otolith measurements in male and female Atlantic cod

Females				Males		
	Low *p*CO_2_ (N = 34)	High *p*CO_2_ (N = 38)	Diff (%)	Low *p*CO_2_ (N = 47)	High *p*CO_2_ (N = 60)	Diff (%)
*Bw (g)*	7180 ± 1720	7688 ± 1835		6285 ± 1286	6745 ± 1492	
*SL (cm)*	84.23 ± 6.77	89.24 ± 8.97		82.09 ± 4.67	84.30 ± 6.60	
*OW (mg)*	0.78 ± 0.15 (***0.80* ± *0.01***)*	0.83 ± 0.14 (***0.85* ± *0.02***)	+ 6	740 ± 113 (***0.75* ± *0.01***)	774 ± 148 (***0.77* ± *0.01***)	+ 2.5
*OL (mm)*	20.68 ± 1.67 (***21.5* ± *0.23***)*	20.53 ± 1.40 (***20.3* ± *0.20***)	− 5.6	19.63 ± 1.01 (***19.7* ± *0.18***)	19.87 ± 1.44 (***19.8* ± *0.15***)	+ 0.5
*OR (mm)*	10.12 ± 0.56 (***10.3* ± *0.09***)	10.18 ± 0.59 (***10.1* ± *0.08***)	− 2	9.65 ± 0.63 (***9.73* ± *0.09***)	9.99 ± 0.76 (***9.95* ± *0.07***)	+ 2.3
*OP (mm)*	53.19 ± 4.06 (***54.3* ± *0.57***)*	52.65 ± 2.77 (***52.4* ± *0.51***)	− 3.5	50.76 ± 2.60 (***51.0* ± *0.45***)	51.03 ± 3.53 (***50.9* ± *0.37***)	+ 0
*OA (mm*^*2*^*)*	160.69 ± 31.30 (***168* ± *2.80***)*	161.27 ± 16.50 (***160* ± *2.51***)	− 4.8	146.42 ± 15.39 (***148* ± *2.39***)	153.46 ± 20.46 (***152* ± *1.94***)	+ 4.6
*OD*	4.89 ± 0.45 (***5.05* ± *0.04***)*	5.20 ± 0.42 (***5.26* ± *0.04***)	+ 4	5.02 ± 0.35 (***4.99* ± *0.04***)	5.02 ± 0.45 (***5.08* ± *0.03***)	+ 1.8
*Cicl*	17.60 ± 1.38 (***17.7* ± *0.24***)	17.19 ± 1.32 (***17.3* ± *0.22***)	− 2.3	17.59 ± 1.45 (***17.5* ± *0.20***)*	16.97 ± 1.18 (***17.0* ± *0.16***)	− 3
*Rd*	0.48 ± 0.02 (***0.47* ± *0.005***)	0.49 ± 0.03 (***0.49* ± *0.005***)	+ 4	0.48 ± 0.03 (***0.48* ± *0.005***)*	0.49 ± 0.03 (***0.50* ± *0.003***)	+ 4
*Rect*	0.76 ± 0.02 (***0.77* ± *0.005***)	0.77 ± 0.03 (***0.77* ± *0.005***)	+ 0	0.77 ± 0.02 (***0.77* ± *0.003***)	0.77 ± 0.02 (***0.77* ± *0.003***)	+ 0
*Ellip*	0.34 ± 0.02 (***0.34* ± *0.004***)	0.33 ± 0.02 (***0.33* ± *0.004***)	− 3	0.33 ± 0.03 (***0.34* ± *0.005***)	0.32 ± 0.03 (***0.33* ± *0.004***)	− 3

Average values and adjusted means (in brackets) of female and male Atlantic cod exposed to 429 and 1091µatm *p*CO_2_ for *Bw* body weight, *SL* standard length and *OW* otolith weight, *OL* otolith length, *OR* otolith radius, *OP* otolith perimeter, *OA* otolith area, *OD* = *OW/OA* otolith density, *Cicl* circularity, *Rd* roundness, *Rect* rectangularity and *Ellip* ellipticity. Unadjusted data is expressed as Mean ± SD and adjusted means as least squares ± SE. Differences (Diff) are expressed as a percentage of the differences between adjusted means. The number of individual otoliths per group is indicated (*N*)

Significant differences between groups for the *pCO*_*2*_ treatment are indicated with an asterisk when *P* < 0.05 (actual *P*-values are in Supplementary File 4)

### Otolith morphology

When visually inspected, all otoliths showed the characteristic shape associated to Atlantic cod (McBride et al. 2010), and 90% of them had a smooth and homogeneous white surface (Supplementary File 1). However, we also found 5 animals from the high *p*CO_2_ group (10% of the total) showing an abnormal deposition covering up to 10% of the surface for one or both otoliths.

**Fig. 1:**
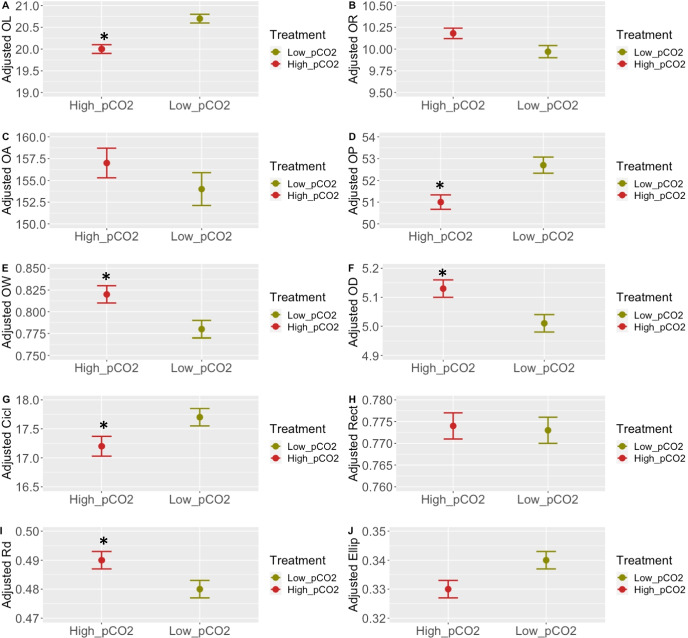
Adjusted means for otolith measurements and shape indexes from Atlantic cod exposed to 1091 (high *p*CO_2_) and 422µatm (low *p*CO_2_) for a period of 30 weeks. Values are expressed as adjusted mean ± SE for 422 (low *p*CO_2_; *N* = 81) (green) and 1091µatm (high *p*CO_2_; N = 98) (red) groups. Asterisks indicate significant differences between treatments (*P < 0.05). Otolith length (*OL*) (**A**), otolith width (*OR*) (**B**), otolith area (*OA*) (**C**), otolith perimeter (*OP*) (**D**), otolith weight (*OW*) (**E**), otolith density (*OD*) (**F**), circularity (*Cicl*) (**G**), rectangularity (*Rect*) (**H**), roundness (*Rd*) (**I**) and ellipticity (*Ellip*) (**J**)

As expected, all otolith measurements were significantly correlated to SL and *OW* (*ρ* = 0.73; *P* < 0.001), *thickness* (*ρ* = 0.50; *P* < 0.001), *OL* (*ρ* = 0.56; *P* < 0.001), *OR* (*ρ* = 0.55; *P* < 0.001), *OP* (*ρ* = 0.50; *P* < 0.001) and *OA* (*ρ* = 0.63; *P* < 0.001) (Supplementary File 2); therefore, SL was included as a covariant in the mixed models to study otolith measurements, and adjusted means were used for comparisons between treatments. Only *OL* and *OP* were significantly affected by the *p*CO_2_ *treatment* (*P* = 0.03 and *P* = 0.04, respectively). Adjusted means of the measurements showed a 3.4% and 3.3% reduction in *OL* and *OP* for individuals of the high *p*CO_2_ group (Fig. 1; Table 2; Supplementary File 3). Otolith weight (*OW*) and density (*OD*) were a 5% and 2.5% higher in animals of the high *p*CO_2_ group (Table 2).

The different *p*CO_2_ treatments also had a significant effect on roundness (*Rd*) (4% increase in high *p*CO_2_ group; *P* = 0.04) and circularity (*Cicl*) (− 2.9% reduction in high *p*CO_2_ group; *P* = 0.03) (Fig. 1; Table 2; Supplementary File 3). We also found that *OW* had a significant effect on *Cicl* and *Rect* (*P* < 0.001 for both cases), but treatment*OW interaction was not significant for any of the shape indexes (Fig. 1; Table 2; Supplementary File 3), suggesting that differences in shape between treatments were not affected by age.

### Effect of animal gender on otolith morphometry and shape

**Fig. 2:**
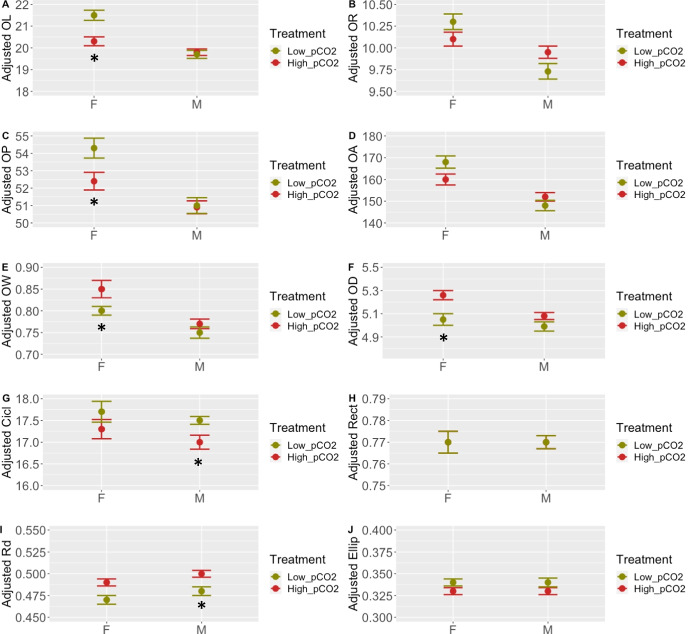
Adjusted means for otolith measurements and shape index from males (M) and females (F) of Atlantic cod exposed to 1091 (high *p*CO_2_; male otoliths *N* = 60, female otoliths *N* = 38) and 422µatm (low *p*CO_2_; male otoliths *N* = 47, female otoliths *N* = 34) for a period of 30 weeks. Asterisks indicate significant differences between treatments (*P < 0.05). Otolith length (*OL*) (**A**), otolith width (*OR*) (**B**), otolith area (*OA*) (**C**), otolith perimeter (*OP*) (**D**), otolith weight (*OW*) (**E**), otolith density (*OD*) (**F**), circularity (*Cicl*) (**G**), rectangularity (*Rect*) (**H**), roundness (*Rd*) (**I**) and ellipticity (*Ellip*) (**J**)

*OW*, *OL*, *OR*, *OP*, *OA*, and *OD* parameters had a significant gender*treatment interaction (Supplementary File 4), indicating a different response to *p*CO_2_ treatments between males and females. To determine the existence of any gender-specific susceptibility, the data was re-analysed separately for males and females (Fig. 2; Table 3; Supplementary File 4 and 5).

Again, all otolith measurements strongly correlated to SL for both genders (Supplementary File 5), and *SL* was included as a co-variant in the models. We found that females from the high *p*CO_2_ treatment had smaller (*OL*, *OP* and *OA* were − 5.6, − 3.5 and − 4.8% smaller; *P* = 0.03, 0.03 and 0.04, respectively) and heavier (*OW* and *OD* increased + 6% and + 4%; *P* = 0.03 and *P* = 0.02, respectively) otoliths than females of the low *p*CO_2_ group (Fig. 2; Table 3, Supplementary File 4).

The *p*CO_2_ levels had a significant effect on high *p*CO_2_ males’ otolith *Cicl* and Rd (− 3% and + 4% changes, respectively; *P* = 0.03 and 0.04) compared to males of the low *p*CO_2_ group (Table 3; Fig. 2; Supplementary File 4). No significant changes were found in males’ otolith dimensions.

*OW* (as a proxy for age) significantly influenced shape indexes (*Cicl*, *Rect* and *Round* on males and *Rect* and *Round* in females) (Supplementary File 4), suggesting that age had an overall effect on otolith shape, as we would expect from a mixture of animals of different ages. However, the treatment*OW interaction was not significant for any of the indexes (Supplementary File 4), suggesting that age does not explain the differences found between treatments.

### CaCO_3_ polymorph identification

**Fig. 3:**
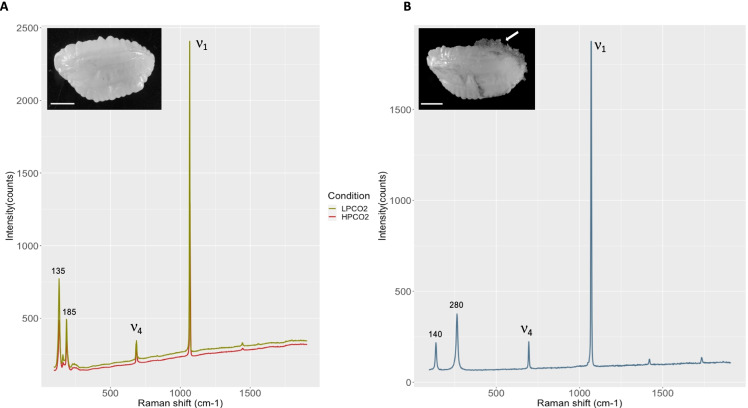
Raman spectrometry on cod otoliths exposed to 1091 and 422µatm *p*CO_2_ for a period of 30 weeks. Aragonite Raman reading obtained from high *p*CO_2_ (red) and low *p*CO_2_ (green) normal otoliths (**A**). Raman reading obtained from an abnormal deposition found in 10% of the high *p*CO_2_ individuals (**B**) showing a typical calcite profile. Calcium carbonate lattice ν1 and ν4 peaks are indicated, and the shift positions of relevant peaks are indicated. Scale bar indicates 5 mm

Raman readings were obtained from the surface of 5 otoliths from each condition. All readings showed the characteristic Raman shifts corresponding to the *ν*_1_ and *ν*_4_ vibrational modes of the CaCO_3_ lattice (1070 cm^−1^, 680–690 cm^−1^ and 135 cm^−1^) together with a peak at 190 cm^−1^ specific to aragonite (Fig. 3). Raman measurements were also taken from the abnormal depositions identified in 10% of the high *p*CO_2_ individuals showing a characteristic calcite profile with the common peaks of the CaCO_3_ lattice and a calcite-specific peak at 280 cm^−1^ (Fig. 3).

## Discussion

In the present study, we found indications that exposure of adult Atlantic cod to 1091µatm *p*CO_2_ for 30 weeks had a significant, but gender-specific, effect on otolith morphology. Otoliths from females exposed to high *p*CO_2_ treatment (High *p*CO_2_) were relatively smaller compared to low *p*CO_2_ females (a reduction of 2–6% for *OL*, *OR*, *OP* and *OA* in females from the high *p*CO_2_), while otoliths from males exposed to the high *p*CO_2_ treatment appeared slightly rounder (− 3% in *Cicl* and + 4% in *Rd*) when compared to those from the low *p*CO_2_ group (Supplementary File 6). Our observations contrast with previous studies on fish larvae where either an increase or no change in otolith size was observed in response to high *p*CO_2_ such as for Atlantic cod (Maneja et al. 2013), cobia (Bignami et al. 2013), Baltic cod (Frommel et al. 2013) or gilthead sea bream (Coll-Lladó et al. 2018). However, previous studies have not normally distinguished between males and females in those species where it is possible.

There is no conclusive explanation for the different gender susceptibility to high *p*CO_2_ observed in the present study. There is the possibility that it might be linked to energy budget constraints between growth, development and acid–base regulation. Otolith growth, like somatic growth, is limited by the energy availability (Fablet et al. 2011), and increased energy expenditure to counteract ocean acidification effects may hamper otolith formation. It is known that cod females have higher energy demands for gonad development than males (Gjedrem 2000; Karlsen et al. 1995). Changes in otolith structure (opacity pattern) are observed in some species in response to spawning (Katayama 2018) likely as a result of the energy budget constraints during that period (Irgens 2018). Similarly, differences in otolith growth are observed in fish species that change gender during their lifecycle, linked to changes in growth rate (Walker and McCormick 2009). Animals used in the present study (caught in October) spawned during March of the following year, and their weight was significantly reduced during that period, but this reduction was more prominent in females from the high *p*CO_2_ group (Supplementary File 7). Since females had a higher energy demand for gonadal development and egg production, the energy budget of high *p*CO_2_ females would have been more constrained trying to maintain the acid–base balance, reducing somatic growth capacity and, consequently, otolith growth when compared to low *p*CO_2_ females. In addition to changes in size, we found a significant increase in average otolith weight (+ 6%; *P* = 0.04) and density (+ 4%; *P* = 0.02) of otoliths (using *OW/OA* as a proxy) in females and also, but not significantly, in males exposed to high *p*CO_2_ levels (Fig. 2; Table 3). These results are in agreement with previous studies in which an increase in otolith density was observed in response to higher *p*CO_2_ (Bignami 2013). It has also been suggested that endolymph *p*CO_2_ and HCO_3_ increase when exposed to ocean acidification conditions in parallel to plasma levels, leading to enhanced carbonate deposition in the otoliths of some species (Grosell 2019). The increase in otolith density seems to indicate that there is an increase in CaCO_3_ accretion that is not translated into larger otoliths. One possible hypothesis is that while the CaCO_3_ fraction of the otolith was increasing, the protein fraction in adult cod decreased as a result of changes in acid/base physiology, although this possibility needs to be further investigated. The present results should be considered as a pilot study since important information from a sampling before the experimental period was not performed. Furthermore, the animals were not treated with calcein or any similar dye to determine how much CaCO_3_ was deposited during the experimental period between the two experimental groups.

Otolith shape indexes are not commonly assessed in response to ocean acidification, and just a few xMunday et al. 2011; Réveillac et al. 2015) or no effects (Checkley et al. 2009b) have been reported. The otolith shape, proportions and lobe number in Atlantic cod have a genetic component but, together with the otolith weight, they are also affected by age and environmental factors, such as temperature or diet (e.g. Mille et al. 2016; Vignon and Morat 2010). Otolith parameters in cod, such as otolith weight and number of lobes, can rapidly change during the first years of life (0 to 3 years) but at a much slower rate in the following years (Campana and Fowler 2012; Irgens 2018). In the present study we used the otolith weight (*OW*) as a proxy for the animal’s age, and their values ranged from a minimum of 0.5 to a maximum of 1.02 g (average 0.75–0.80 g), indicating age diversity as it could be expected for wild populations. Statistical analysis showed that *OW* had a significant effect on the majority of shape indexes analysed, suggesting they were influenced by age. However, OW*treatment interaction was not significant in any case, and, therefore, differences observed between groups were independent of age. This is possibly due to the similar *OW*/age distribution between the groups (Supplementary File 2 and 5). Studies based on mathematical models already suggested that larger otoliths would generate sound wave displacement with a possible detrimental effect on hearing (Bignami et al. 2013). A recent study by Radford et al. (2021) has confirmed some of the predictions from these models, showing that otolith size and fluctuating asymmetry under conditions of ocean acidification cause a decline in low frequency hearing in fish (Radford et al. 2021). It is possible that if important changes in the shape are not followed by modifications in the inner ear morphology, this might lead to physical constraints between the two compartments and have consequences on sound propagation and hearing sensitivities. Changes observed in the present study were minor but raise the possibility that chronic exposure to high *p*CO_2_ would produce larger differences. However, more detailed studies about the relationship between otolith shape and inner ear morphology in response to ocean acidification and in vivo analysis of hearing capabilities are needed.

Otolith formation is strongly dependent on the endolymph composition. The growth of the otolith reflects both the aragonite saturation state (Ω) of the endolymph (indicating the concentration of ${\mathrm{CO}}_{3}^{-2}$ and Ca^+2^ available to produce the mineral phase) and its organic composition (Payan et al. 2004). Dissolved inorganic carbon occurs as H_2_CO_3_, ${\mathrm{HCO}}_{3}^{-}$ and ${\mathrm{CO}}_{3}^{-2}$, with the formation of ${\mathrm{CO}}_{3}^{-2}$ favoured at high pH. Proton secretion from the endolymph serves to increase endolymph pH and likely promotes aragonite formation (Payan et al. 1998). In the vast majority of otoliths examined, aragonite was the predominant CaCO_3_ polymorph; however, approximately 10% of the high *p*CO_2_ fish group showed calcite deposition on the otolith surface (Fig. 3). Although very rare, aragonite replacement by calcite can occur spontaneously in wild populations (Oliveira et al. 1996), but the reasons are still unknown. Some authors have suggested that aragonite can be replaced by other polymorphs such as vaterite when growth rates are increased (Reimer et al. 2017) or animal density is high (Austad et al. 2021). While possible changes in growth rate might be the reason for calcite deposition, the fact that only 10% of the animals were affected makes that assumption unlikely. Under normal circumstances, the endolymph is more alkaline (pH = 8.0) than the plasma (pH = 7.2–7.6), partly determined by the concentration of bicarbonate and the relatively high levels of CO_2_ in the endolymph (30 mmol^−1^) compared to the plasma (8–12 mmol^−1^) (Campana 1999; Payan et al. 2004). Studies in vitro have shown that CaCO_3_ precipitates as calcite when the pH of the media increases (Ren et al. 2013). It is possible that the increase of ${\mathrm{HCO}}_{3}^{-}$ and *p*CO_2_ in the high *p*CO_2_ animals could increase the alkalinity of the saccular endolymph beyond a certain threshold that would facilitate the replacement of aragonite by calcite. Another possibility might be potential changes in the protein matrix composition driving this polymorph replacement. Previous studies have shown that the otolith’s organic matrix plays a crucial role in carbonate crystallization (Falini et al. 2005; Ren et al. 2013), so if the protein matrix composition is altered by more acidic pH, that might lead to calcite deposition. Hughes et al. (2004) reported that abnormal calcite otoliths are formed in zebrafish when *otopetrin-1* was inhibited in knockout fish, demonstrating the importance of the protein matrix for otolith formation (Hughes et al. 2004). However, since only 10% of the individuals in our study appeared to be affected, it might be an indication of genetic susceptibility of some individuals to deposit calcite in response to high *p*CO_2_. Calcite deposition has also been observed in 21% of gilthead sea bream larval otoliths when exposed to 2000µatm of *p*CO_2_ (Coll-Lladó et al. 2018), and this was attributed to a significant degree of heritability (*h*^2^ = 0.44–0.55%), suggesting genetic susceptibility to calcite deposition. Although it is possible that Atlantic cod showing calcite deposition were genetically more susceptible to crystallize calcite in response to high *p*CO_2_, we cannot rule out other factors, and, therefore, further research is needed to confirm this hypothesis.

While the current study has some limitations, the results obtained give us some perspective of how ocean acidification might impact adult fish. Some of the limitations include the difficulty to replicate treatments due to welfare considerations, so despite keeping equal environmental parameters (such as light, temperature and diet) between tanks, we cannot rule out the tank effect; although most of the studies in which a tank effect has been observed, this was attributable to environmental or treatment differences (Speare et al. 1995). Also, to overcome the intrinsic variability present on wild populations, we did not perform a pre-experimental sampling in order to have enough statistical power; therefore, the information about the otoliths before starting the experimental trial is lacking. Finally, wild populations have a mixture of animals of different ages, but empirical estimation of cod’s age by counting the annual growth increments was not performed and otolith weight (*OW*) was used as a proxy for age since both parameters correlate well (Bermejo 2014; Campana and Fowler 2012; Pawson 2006). In the present study, growth rates were similar between groups, with a significant weight reduction during the spawning occurred during ~ 24 weeks after capture (Supplementary File 5). Since no significant differences in weight, standard length or *OW* (as estimation of age) were found between the two experimental groups, we believe the differences between otolith parameters between the two groups were likely determined by the effect of *p*CO_2_.

## Conclusions

Exposure of adult cod to 1091µatm of *p*CO_2_ for a period of 30 weeks had a relatively small but significant effect on otolith size and shape in a gender-dependent way. Males exposed to 1091µatm had rounder otoliths whereas female otoliths were smaller. We also found calcite aggregations in 10% of the animals exposed to 1091µatm *p*CO_2_, suggesting individual susceptibility to deposit calcite. Despite the experimental limitations of the present pilot study, our results suggest that even when otoliths are well formed and during periods of slow growth (such as in adults), exposure to high *p*CO_2_ still has a significant effect on their formation.

## Supplementary Information

Below is the link to the electronic supplementary material.

Supplementary file1 (DOCX 5074 KB)

Supplementary file2 (PDF 375 KB)

Supplementary file3 (DOCX 16 KB)

Supplementary file4 (DOCX 18 KB)

Supplementary file5 (PDF 417 KB)

Supplementary file6 (PDF 331 KB)

Supplementary file7 (PDF 156 KB)

## Data Availability

The datasets generated during and/or analysed during the current study are available from the corresponding author on reasonable request.
