# Supplementary material for: Pilot study to investigate the effect of long-term exposure to high pCO2 on adult cod (Gadus morhua) otolith morphology and calcium carbonate deposition
Source: Fish Physiol Biochem. 2021 Sep 28;47(6):1879–91. doi: 10.1007/s10695-021-01016-6 (PMC8636414; doi:10.1007/s10695-021-01016-6)
Supplement: Supplementary file 1 — Supplementary file1 (DOCX 5074 KB) [file 10695_2021_1016_MOESM1_ESM.docx]

**Supplementary File 1. Adult Cod Otolith with schematic representation of measurements taken.**

Standard image taken from Atlantic cod otolith. Main measurements taken, otolith orientation and regions are indicated. Scale bar represents 5mm.

OL: Otolith Length.

OR: Otolith Width.
