# Supplementary figures and images for: Pilot study to investigate the effect of long-term exposure to high pCO2 on adult cod (Gadus morhua) otolith morphology and calcium carbonate deposition
Source: Fish Physiol Biochem. 2021 Sep 28;47(6):1879–91. doi: 10.1007/s10695-021-01016-6 (PMC8636414; doi:10.1007/s10695-021-01016-6)

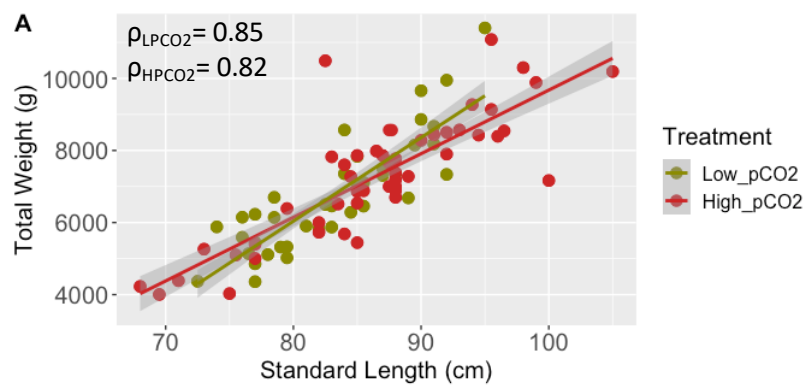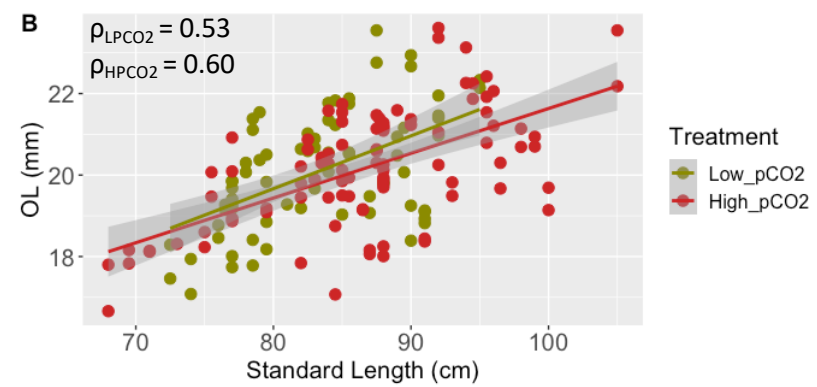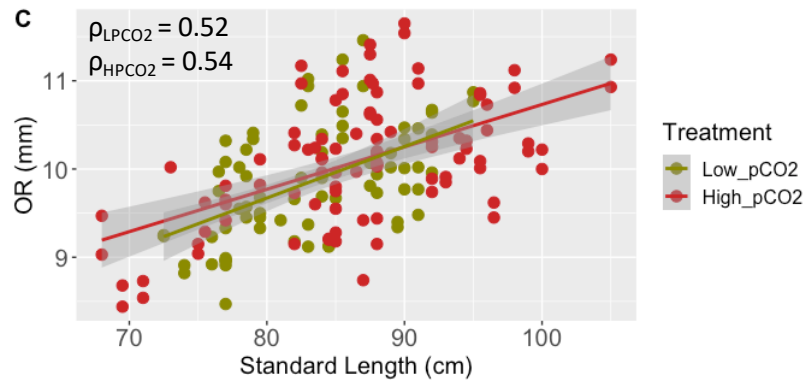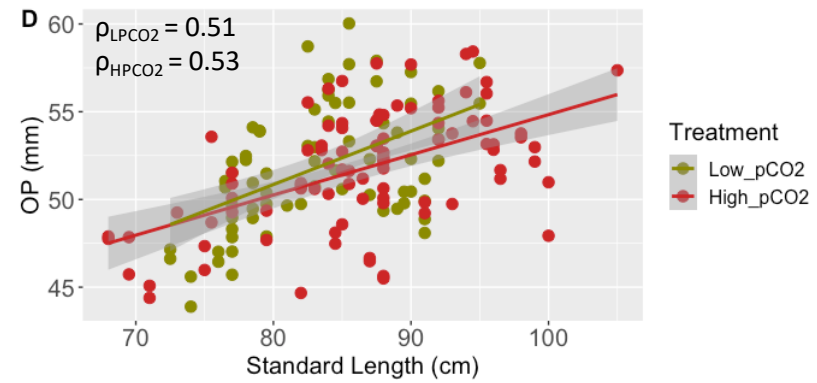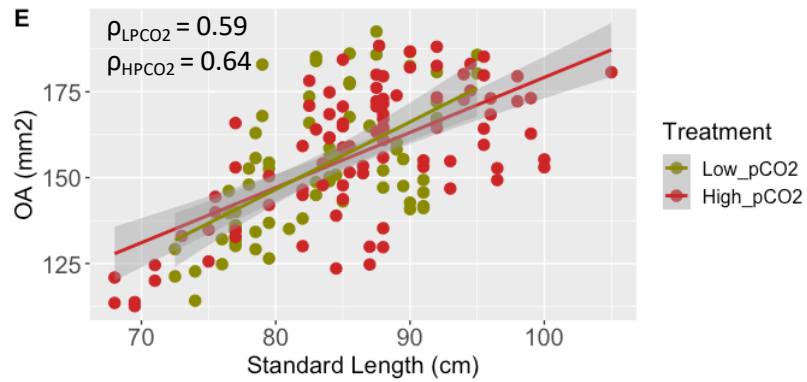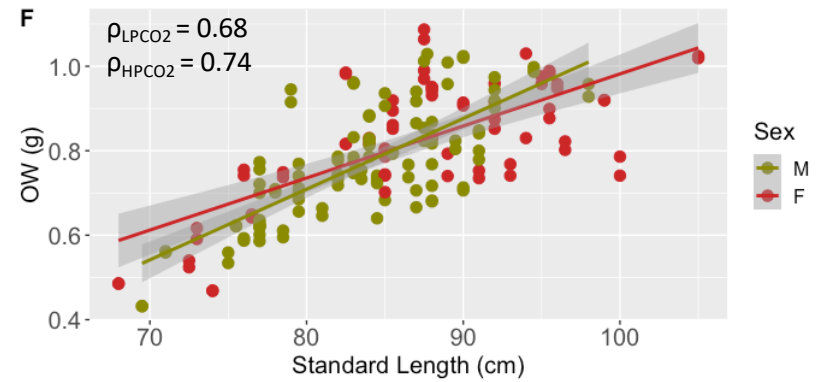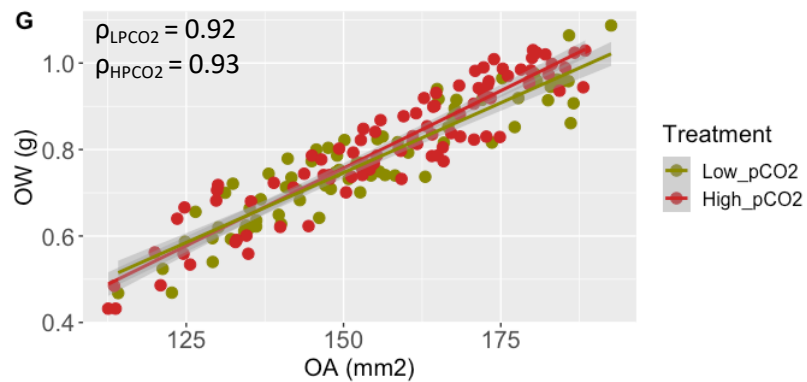

Supplement: Supplementary file 2 — Supplementary file2 (PDF 375 KB) [file 10695_2021_1016_MOESM2_ESM.pdf]

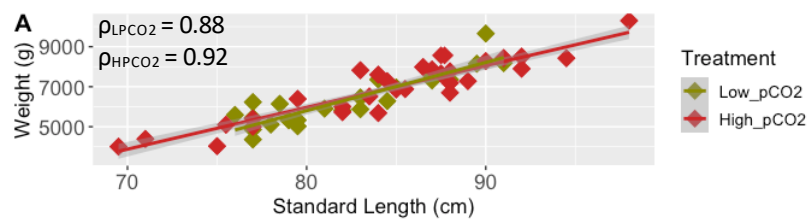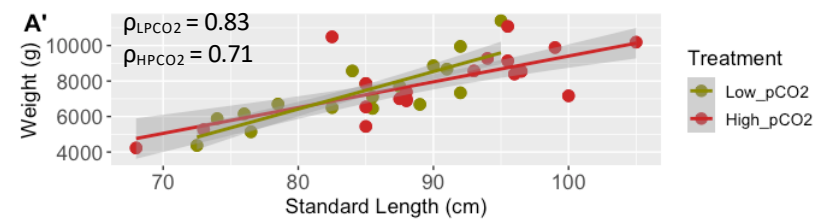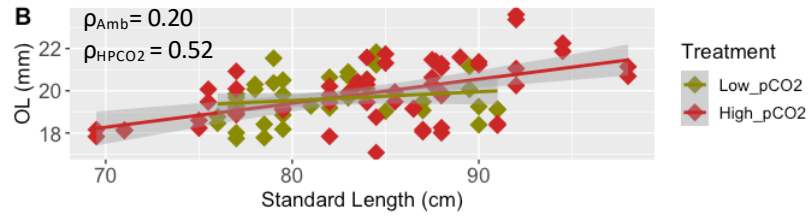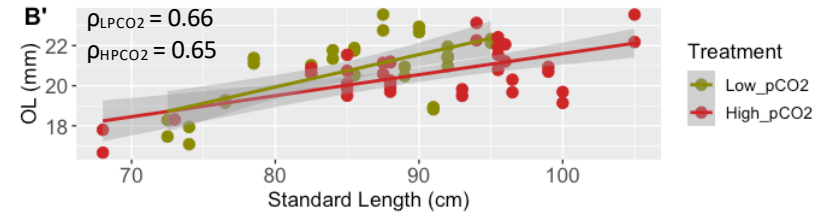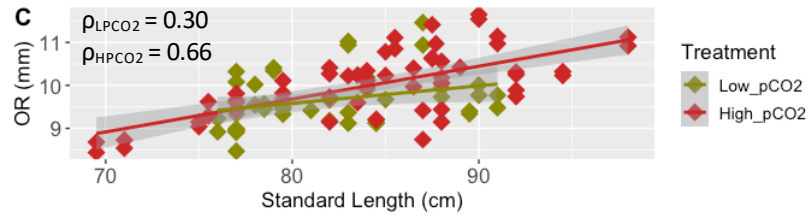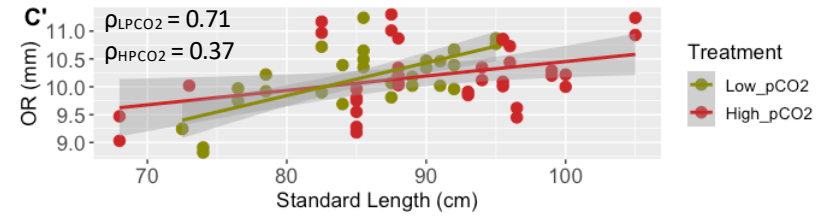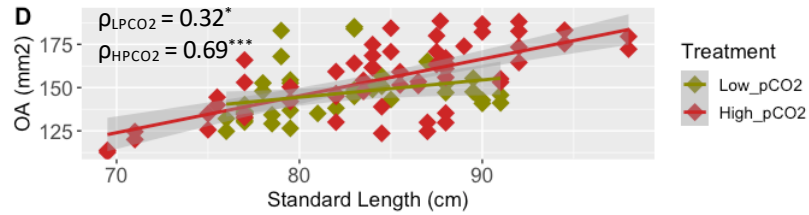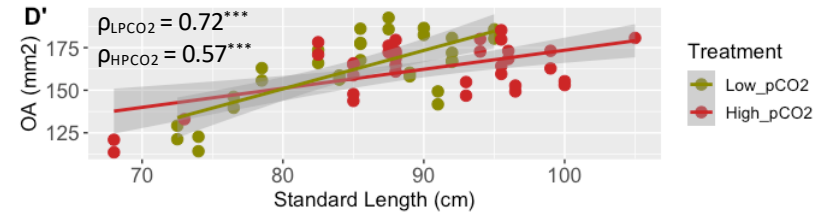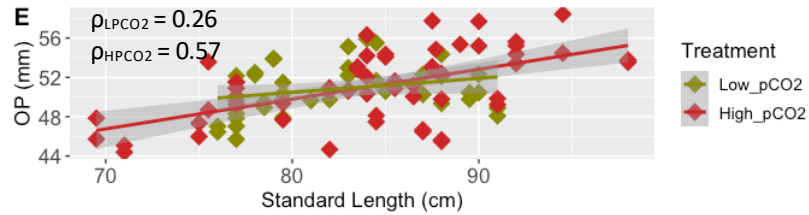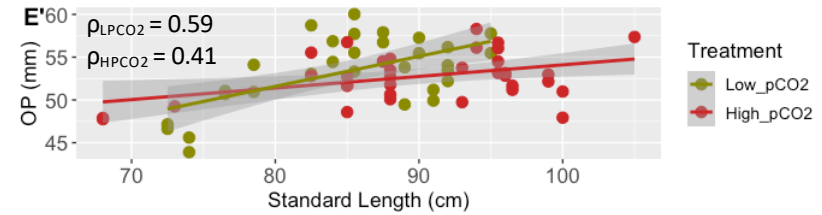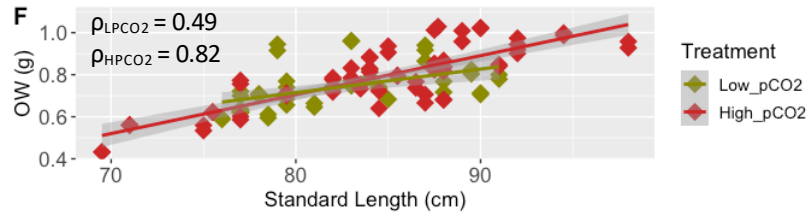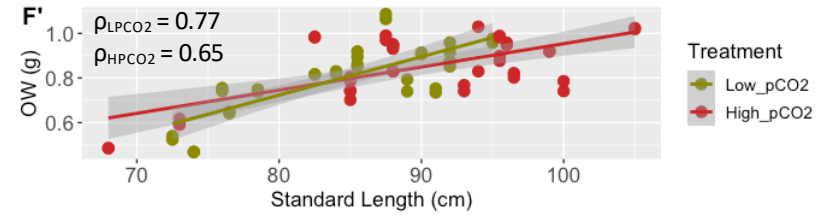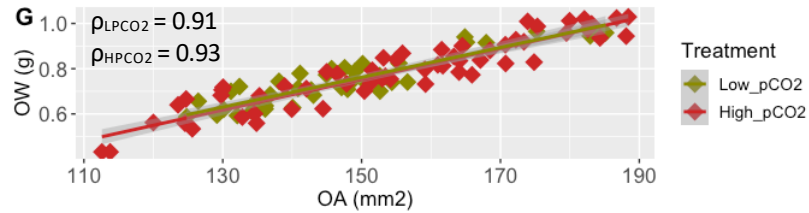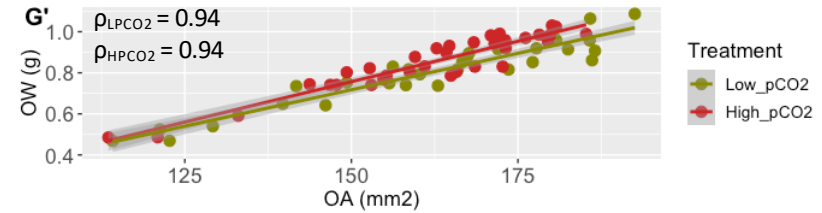

Supplement: Supplementary file 5 — Supplementary file5 (PDF 417 KB) [file 10695_2021_1016_MOESM5_ESM.pdf]

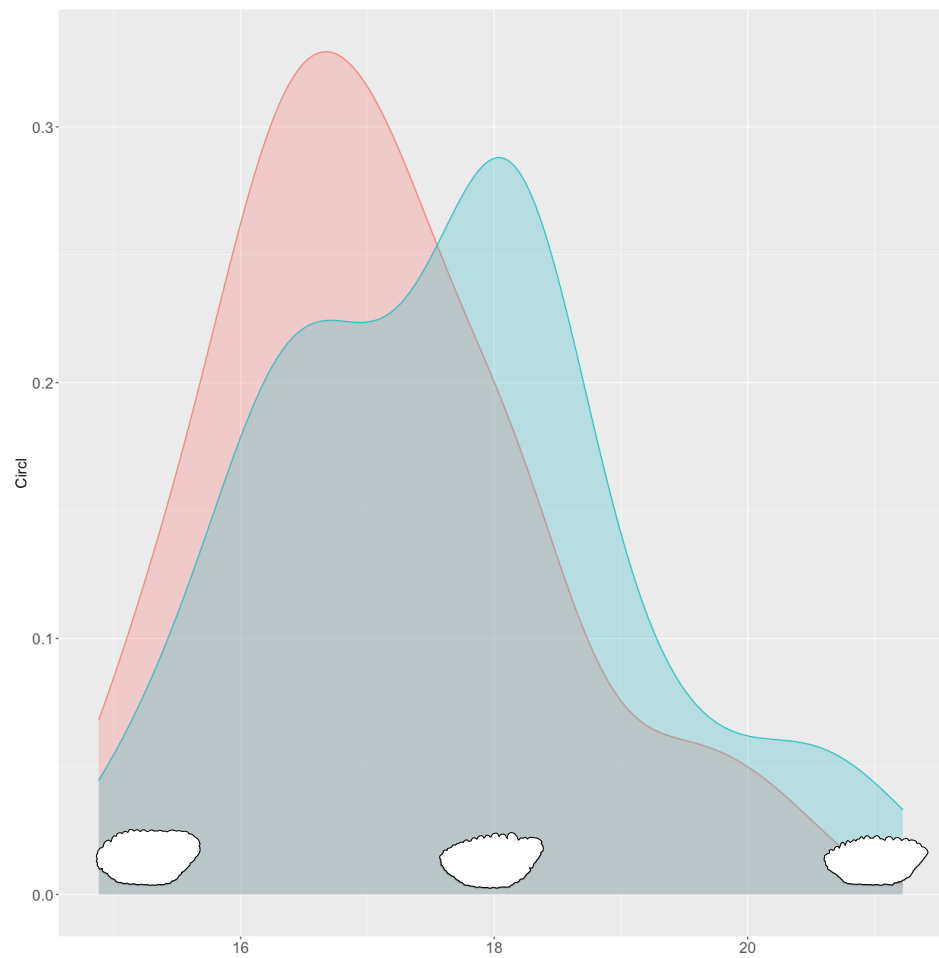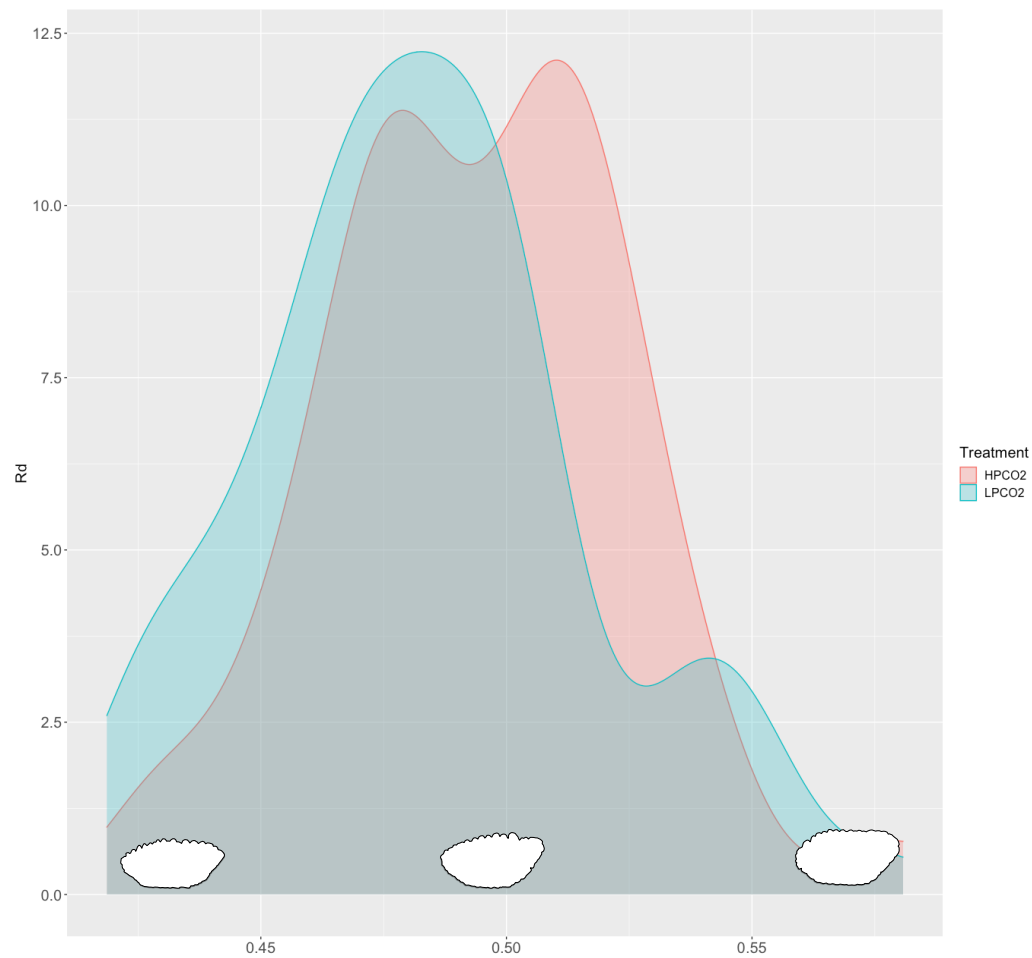

Supplement: Supplementary file 6 — Supplementary file6 (PDF 331 KB) [file 10695_2021_1016_MOESM6_ESM.pdf]

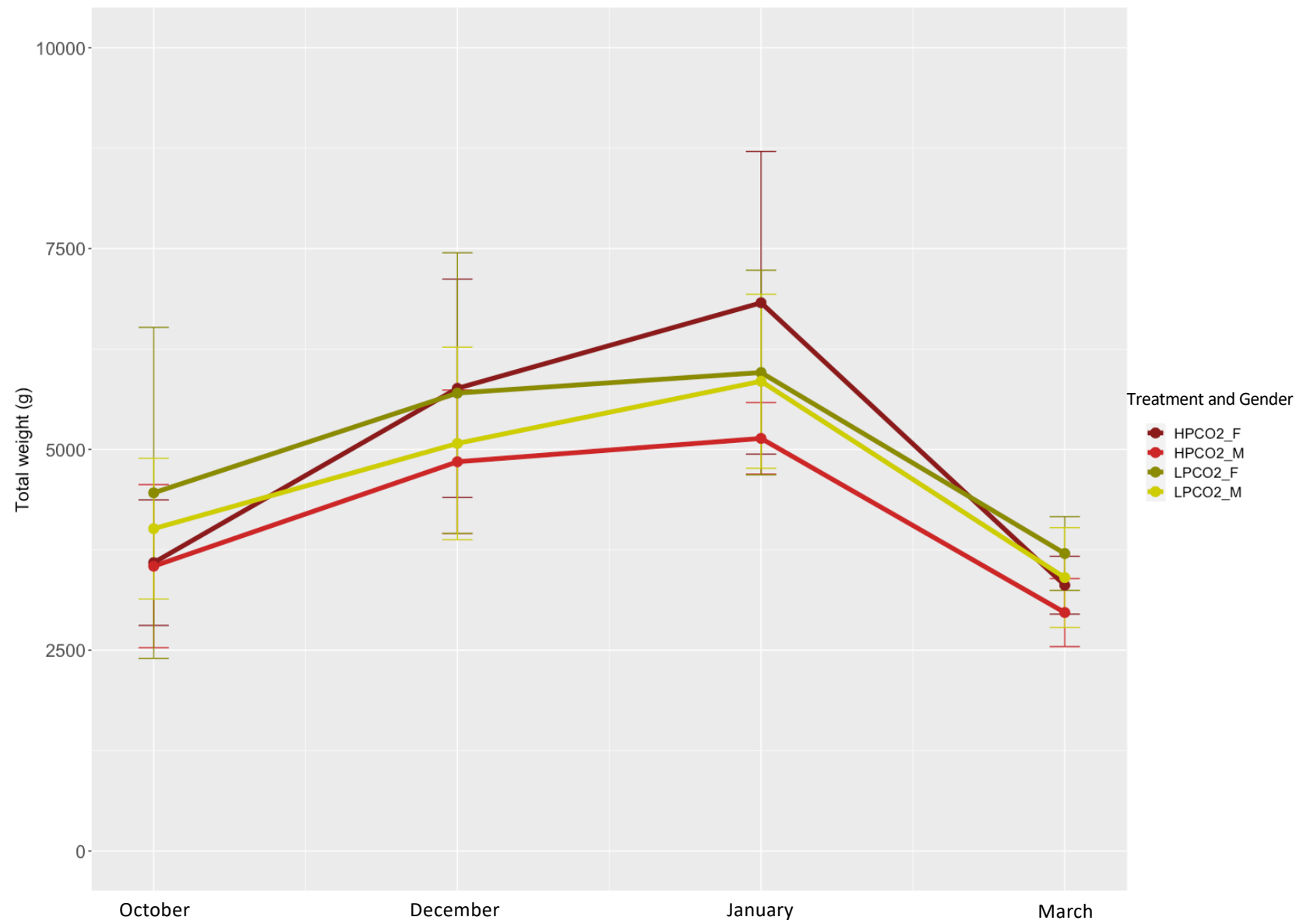

Supplement: Supplementary file 7 — Supplementary file7 (PDF 156 KB) [file 10695_2021_1016_MOESM7_ESM.pdf]
