# Supplementary material for: Pilot study to investigate the effect of long-term exposure to high pCO2 on adult cod (Gadus morhua) otolith morphology and calcium carbonate deposition
Source: Fish Physiol Biochem. 2021 Sep 28;47(6):1879–91. doi: 10.1007/s10695-021-01016-6 (PMC8636414; doi:10.1007/s10695-021-01016-6)
Supplement: Supplementary file 3 — Supplementary file3 (DOCX 16 KB) [file 10695_2021_1016_MOESM3_ESM.docx]

**Supplementary File 3.** Statistical analysis (P-values) of *standard* *length*, *p*CO_2_ *treatment, OW* and *animal gender* effect on Atlantic cod otolith measurements and shape indexes.

|  | *SL* | *pCO_2_* | *pCO^2^*SL* | *OW* | *gender* | *pCO *gender* | *pCO *OW* | |
| --- | --- | --- | --- | --- | --- | --- | --- | --- |
| *OW* | **0.00** | **0.05** | **0.02** | - | **0.02** | **0.03** |  |  |
| *OL* | **0.00** | **0.05** | **0.13** | **-** | 0.77 | **0.04** |  |  |
| *OR* | **0.00** | 0.09 | 0.07 | - | **0.01** | **0.02** |  |  |
| *OP* | **0.00** | **0.04** | **0.02** | **-** | **0.03** | **0.01** |  |  |
| *OA* | **0.00** | 0.06 | **0.02** | - | **0.01** | **0.01** |  |  |
| *Cicl* | - | **0.04** |  | **0.01** | 0.23 | 0.56 | 0.74 |  |
| *Rect* | - | **0.09** |  | **0.00** | 0.32 | 0.36 | 0.30 |  |
| *Round* | - | **0.04** |  | 0.34 | 0.22 | 0.79 | 0.44 |  |
| *Ellip* | - | 0.07 |  | 0.40 | 0.54 | 0.80 | 0.80 |  |
| *OD* | - | **0.02** |  | - | **0.01** | **0.02** | - |  |

P-values obtained from linear mixed models with *treatment*, *fish length,* *gender* and *OW* (as factors or co-variants) and *head side* (as a random factor). All columns represent p-values and significant estimates are indicated in bold.

*OW*: otolith weight; *OL*: otolith length; *OR*: otolith width; *OA*: otolith area; *OP*: otolith perimeter; *OD:* otolith density (OW/OA); *Cicl*: Circularity; *Rect*: Rectangularity; *Round*: Roundness and *Ellip*: Ellipticity.
