# Supplementary material for: Pilot study to investigate the effect of long-term exposure to high pCO2 on adult cod (Gadus morhua) otolith morphology and calcium carbonate deposition
Source: Fish Physiol Biochem. 2021 Sep 28;47(6):1879–91. doi: 10.1007/s10695-021-01016-6 (PMC8636414; doi:10.1007/s10695-021-01016-6)
Supplement: Supplementary file 4 — Supplementary file4 (DOCX 18 KB) [file 10695_2021_1016_MOESM4_ESM.docx]

**Supplementary File 4.** Statistical analysis (P-values) of *standard* *length (SL)*, *p*CO_2_ *treatment* and *OW* effect on otolith measurements and shape indexes from female and male Atlantic cod.

| ***Males*** | *SL* | *pCO_2_* | *pCO_2_*SL* | *OW* | *pCO_2_*OW* |
| --- | --- | --- | --- | --- | --- |
| *OW* | **0.00** | 0.06 | **0.03** | - | - |
| *OL* | **0.00** | 0.10 | 0.10 | - | - |
| *OR* | **0.00** | 0.14 | 0.12 | - | - |
| *OP* | **0.00** | 0.12 | 0.13 | - | - |
| *OA* | **0.00** | 0.08 | 0.05 | - | - |
| *Cicl* | - | **0.03** | **-** | **0.00** | 0.16 |
| *Rect* | - | 0.13 | **-** | **0.00** | 0.11 |
| *Round* | - | **0.04** | **-** | **0.00** | 0.16 |
| *Ellip* | - | 0.25 | - | 0.21 | 0.58 |
| *OD* | **0.00** | 0.33 | 0.18 | - | - |
|  |  |  |  |  |  |
| ***Females*** | *SL* | *pCO_2_* | *pCO_2_*SL* | *OW* | *pCO_2_*OW* |
| *OW* | **0.00** | **0.04** | **0.04** | - | - |
| *OL* | **0.00** | **0.03** | 0.14 | - | - |
| *OR* | **0.00** | 0.06 | **0.04** | - | - |
| *OP* | **0.00** | **0.03** | **0.03** | - | - |
| *OA* | **0.00** | **0.04** | **0.02** | - | - |
| *Cicl* | - | 0.12 | - | 0.10 | 0.10 |
| *Rect* | - | 0.25 | **-** | **0.05** | 0.30 |
| *Round* | - | 0.30 | **-** | **0.05** | 0.66 |
| *Ellip* | - | 0.43 | **-** | **0.00** | 0.28 |
| *OD* | **0.00** | **0.02** | 0.09 | - | - |

P-values obtained from linear mixed models with *treatment (pCO2)*, standard length (*SL*) and *otolith weight (OW)* (as factors or co-variants) and *head side* (as a random factor). All columns represent p-values and significant differences are indicated in bold.

All columns represent p-values and significant differences are indicated in bold. *OW*: otolith weight; *OL*: otolith length; *OR*: otolith width; *OA*: otolith area; *OP*: otolith perimeter; *OD:* otolith density (OW/OA); *Cicl*: Circularity; *Rect*: Rectangularity; *Round*: Roundness and *Ellip*: Ellipticity.
